# P-1905. Clinical Reasoning in ID Training: A National Needs Assessment of Fellows

**DOI:** 10.1093/ofid/ofaf695.2074

**Published:** 2026-01-11

**Authors:** Darcy Wooten, Miguel A Chavez

**Affiliations:** Washington University in St. Louis, Kirkwood, MO; Washington University in St. Louis, Kirkwood, MO

## Abstract

**Background:**

Clinical reasoning (CR) is an essential skill in infectious diseases (ID); yet few ID fellowship programs offer a formal curriculum in CR. This may contribute to training variability and increased risk of diagnostic and management errors.

**Figure:**
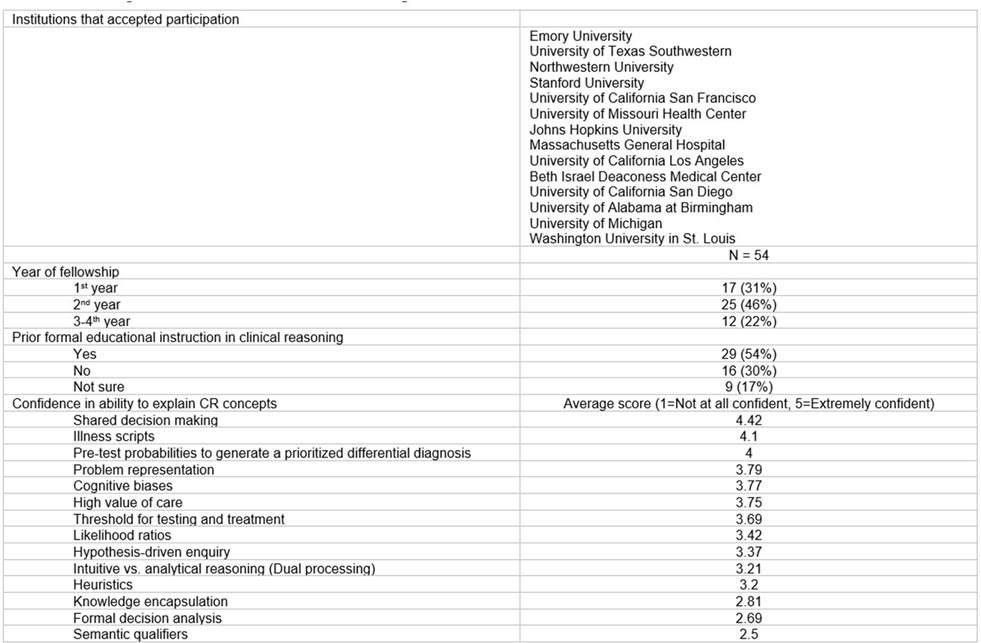
ID fellows’ reported exposure to clinical reasoning (CR) instruction prior to fellowship and self-perceived CR competencies.

**Figure:**
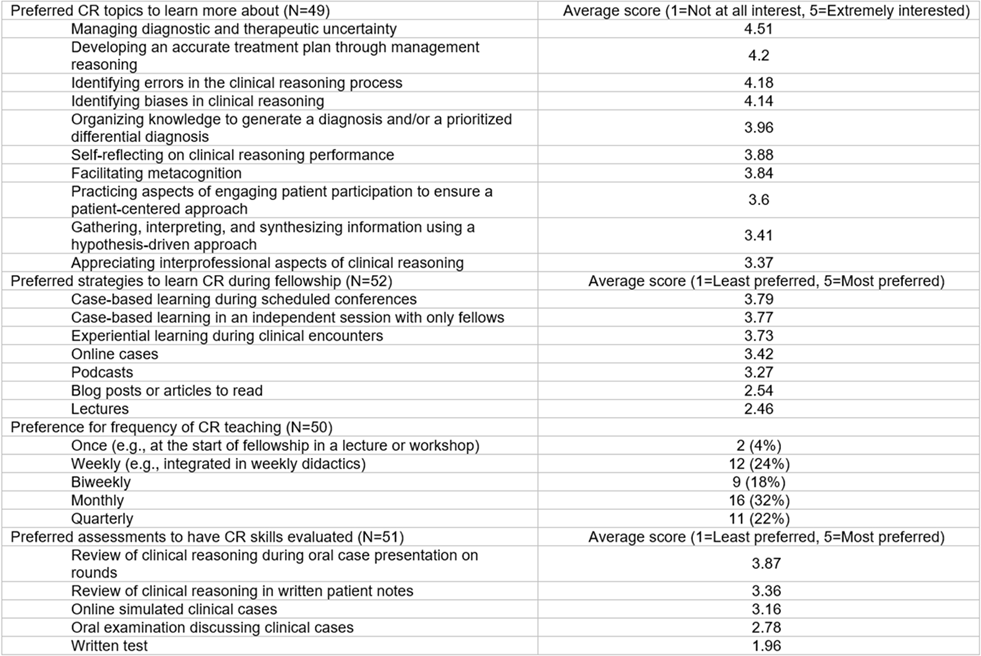
ID Fellows’ reported preferences for CR topics, teaching methods, and assessment tools during fellowship.

**Methods:**

Using convenience sampling, we distributed an anonymous electronic survey to 14 ID fellowship program directors, who were asked to forward it to their fellows. The survey assessed fellows’ prior exposure to CR, self-perceived competencies, preferences for CR teaching methods, and perceived barriers.

**Figure:**
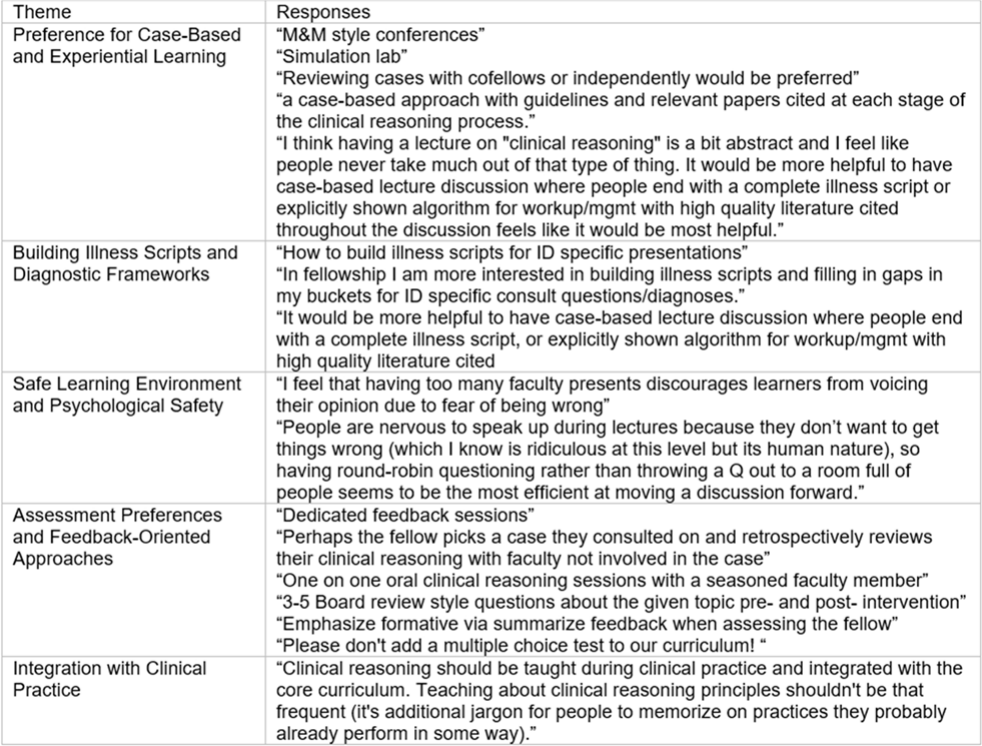
Qualitative input on fellows’ preferences for CR curriculum.

**Results:**

Of 165 fellows, 54 responded (32.7%). Over half (56%) reported prior formal CR instruction (Table 1). Fellows reported the highest confidence in explaining shared decision-making, pre-test probabilities, and illness scripts and the lowest confidence in explaining knowledge encapsulation, decision analysis, and semantic qualifiers. Most (89%) rated having dedicated CR education as moderately to extremely important. While 81% found their current training at least moderately effective for teaching CR, time constraints were cited as the most frequent barrier. Didactic lectures and assigned readings were the least preferred teaching methods for CR, while experiential learning during clinical encounters and case-based learning in conferences or fellow-only sessions were the most preferred (Table 2). Fellows expressed particular interest in learning about managing diagnostic and therapeutic uncertainty, identifying errors in the CR process, developing an accurate treatment plan through management reasoning, and identifying biases. Preferred CR assessment modalities included oral case presentations, written notes, and online simulated cases. Fellows emphasized that case-based instruction on CR and integration of CR teaching into patient care should be prioritized (Table 3). They also highlighted the need for psychological safety and timely, actionable feedback to make CR education effective.

**Conclusion:**

Few fellows receive formal CR instruction prior to fellowship, underscoring a need for intentional integration of CR concepts into ID training. Our findings support the development of a structured CR curriculum with aligned instructional and assessment strategies in ID fellowship programs.

**Disclosures:**

All Authors: No reported disclosures

